# Impact of Regulation on TV White Space Implementation in Brazil: Laboratory and Field Analyses Using 5G-RANGE System

**DOI:** 10.3390/s25082469

**Published:** 2025-04-14

**Authors:** Matheus Sêda Borsato Cunha, Juliano Silveira Ferreira, Anderson Reis Rufino Marins, Rafael Andre Baldo de Lima, Gilberto Zorello, Luciano Leonel Mendes

**Affiliations:** 1 National Institute of Telecommunication, Santa Rita do Sapucaí 37540-000, Brazil; silveira@inatel.br (J.S.F.); anderson@inatel.br (A.R.R.M.); lucianol@inatel.br (L.L.M.); 2 Agência Nacional de Telecomunicações (Anatel), Florianópolis 88010-450, Brazil; rafaell@anatel.gov.br; 3 Núcleo de Informaçao e Coordenação do Ponto BR (NIC.br), São Paulo 04578-000, Brazil; gzorello@nic.br

**Keywords:** 5G-RANGE, DTV, field tests, GFDM, ISDB-T, spectrum coexistence, TVWS

## Abstract

This paper presents the results of field tests conducted in the project “Implementation of TV White Spaces (TVWS) for Internet Access in Brazil”. This study evaluates the feasibility and regulatory implications of TVWS in rural and remote areas. TVWS systems are promising for sensor network applications, enabling efficient and long-range connectivity. The experiments assess the coexistence of TVWS signals, applying, for example, the Remote Area Access Network System for the Fifth Generation (5G-RANGE) using the generalized frequency division multiplexing (GFDM) technique, with the Integrated Services Digital Broadcasting–Terrestrial (ISDB-T) system. Laboratory tests determined the protection ratio (PR) between digital television (DTV) signals and interfering signals, with minimum PR values of −31.38 dB on channel *n*−1 and −33.24 dB on channel n+1 for 5G-RANGE using GFDM, highlighting its low out-of-band emission (OOBE). Field tests confirmed the laboratory results, with the worst recorded PR causing interference being −30.2 dB on channel n−1. The power restriction to 1 Wp limited coverage, allowing 96 Mbps in 24 MHz BW at 14.7 km from the base station. These results highlight that regulatory adjustments can be made to support TVWS deployment in Brazil.

## 1. Introduction

Brazil, being a country with continental dimensions, faces challenges related to limited broadband coverage provided by traditional operators, especially in areas far from large urban centers. Internet access and broadband communications issues act as barriers, leaving individuals in these remote and rural regions digitally excluded due to either poor or expensive services. Furthermore, the high cost of network infrastructure remains a limiting factor for digital inclusion in Brazil [1]. In this context, TV White Space (TVWS) service has emerged as a promising alternative, using available frequencies between TV transmission channels to provide connectivity in hard-to-reach areas. TVWS technology combines its broader spectral coverage with the ability to operate at frequencies that offer greater penetration and range, making it an efficient and cost-effective solution for various applications [2,3]. This feature significantly reduces infrastructure investment, which is key in democratizing high-quality wireless access in Brazil, especially in rural and remote areas with limited telecommunications connectivity. Furthermore, TVWS systems show promising applications in areas such as environmental monitoring, sensor networks for precision agriculture, smart cities, and long-range IoT (LoRa) networks [4,5].

The use of TVWS has been widely studied as a solution to optimize spectrum usage, allowing the coexistence of multiple networks and emerging applications. The IEEE 802.22 standard, designed for cognitive networks with dynamic spectrum access, enables the efficient use of these bands, being particularly promising for vehicular communications [6]. Furthermore, advances in spectral sensing are essential to avoid interference between different systems that share the TVWS, and strategies based on correlation and weight optimization for orthogonal frequency division multiplexing (OFDM) networks have been proposed [7]. In the context of long-range Wi-Fi networks, the IEEE 802.11af standard has been evaluated under realistic propagation conditions, demonstrating potential for rural connectivity and IoT applications [8]. These advances are in line with emerging trends, ranging from the increasing use of fifth-generation of cellular mobile network (5G) technology in Vehicle-to-Everything (V2X) applications for intelligent transportation systems [9], for example, to recent research on sixth generation of cellular mobile network (6G), focusing on the integration of optical and wireless networks [10]. These developments reinforce the need for regulation and implementation of TVWS as a viable means to expand connectivity in Brazil.

Several factors make the commercial exploitation of TVWS in Brazil challenging [11,12]. Observing international experiences, countries like the United Kingdom (UK), the United States of America (USA), Canada, Colombia, and South Africa, for instance, have conducted tests and implementations of TVWS. However, effective exploration of this technology is still in the early stages, partly due to restrictive regulations aimed at protecting primary users [13,14,15]. Regulatory agencies have established guidelines for TVWS usage through standards from the International Telecommunication Union (ITU), the Institute of Electrical and Electronics Engineers (IEEE), and the European Telecommunications Standards Institute (ETSI). To ensure spectrum efficiency, white space device (WSDs) must implement techniques and solutions that prevent interference with primary users, such as spectral sensing and the white space database (WSDB) [16,17,18]. WSDB has been established as the primary method for determining available channels in TVWS. Additionally, other parameters like out-of-band emission (OOBE) attenuation, maximum effective isotropic radiated power (EIRP), power spectral density (PSD) restrictions, and maximum height above average terrain (HAAT) for antennas, which is defined as the height at which the transmission point is above the surrounding topography, are crucial considerations in deploying TVWS services. It is worth noting that while global standardization is desired, these factors may vary according to the country and its regulatory agencies [15,19,20].

The challenges in implementing TVWS, based on the literature, are significant, especially regarding spectrum availability, interference mitigation, and regulatory constraints. One of the main challenges is the accurate identification of available spectrum, as signal attenuation in indoor environments can make it difficult to detect TV white spaces, requiring advanced techniques such as sensing algorithms and geolocation databases. Another critical challenge is mitigating interference with primary services, such as digital television (DTV), particularly due to signal leakage in adjacent channels. Additionally, the spatial and temporal variation of the spectrum poses a problem, as the availability of TVWS channels can change dynamically over time and geographic location. International experience also highlights that spectral fragmentation is an obstacle, as available white spaces are not continuous, requiring flexible radios capable of operating with variable bandwidths. Moreover, the planning of real-world TVWS networks involves additional challenges, such as terrain modeling and adapting equipment to local conditions. In the regulatory context, countries like the USA, UK, Canada, Colombia, and South Africa have faced similar obstacles. While the Federal Communications Commission (FCC) in the USA and Office of Communications (Ofcom) in the UK have allowed unlicensed access to TVWS spectrum, regulation in countries like China and Singapore is still developing, exploring hybrid approaches [3,21,22,23].

The Ofcom, the UK’s communications regulator, has set the maximum EIRP parameter at 36 dBm, which aligns with the standardization by the Federal Communications Commission (FCC) in the USA. Regarding PSD restrictions, Ofcom limits power to 30 dBm over an 8 MHz bandwidth (BW), whereas the FCC specifies the same 30 dBm but over a 6 MHz BW. The BWs refer to the spectral range available for each TV channel in both countries. Concerning OOBE attenuation, the FCC has standardized an attenuation greater than 55.4 dB in adjacent channels. Conversely, Ofcom has traced distinct attenuation values based on device classes, setting a minimum attenuation of 43 dB for Class 5 devices. The HAAT parameter is defined as 250 m by the FCC, whereas Ofcom does not consider the average antenna height to the ground. The Canadian industry adheres to the definitions and standards implemented by the FCC [21,24,25,26,27,28].

The Agencia Nacional del Espectro (ANE) in Colombia has established a maximum output power level delivered to the antenna of 30 dBm and a maximum antenna gain of 14 dBd. The BW of TV channels in Colombia is 6 MHz, and the OOBE attenuation was defined with the same standardized values as the FCC. The maximum HAAT for antenna installation is 800 m. Lastly, the Independent Communications Authority of South Africa (ICASA) regulated parameters, especially focusing on projects using TVWS to expand internet access in rural areas. They set EIRP restrictions for each 8 MHz channel at 41.2 dBm for rural areas and 36 dBm for urban areas. As defined by Ofcom, the values for OOBE attenuations are determined according to device classes, with values greater than 43 dB for Class 5 devices and values greater than 74 dB for Class 1 devices [29,30,31].

Regarding frequency bands designated for TVWS services, there is a predominant focus on standardizing frequencies in the UHF band, but this may vary from country to country. Ofcom defined frequency ranges from 470 MHz to 550 MHz and 614 MHz to 790 MHz, ANE specified frequencies between 400 MHz and 698 MHz, and ICASA standardized the frequency range from 470 MHz to 694 MHz, excluding the sub-band between 606 MHz and 614 MHz intended for Radio Astronomy. North American countries also included Very-High-Frequency (VHF) frequencies in their standardization. The United States set frequency ranges from 54 MHz to 72 MHz, 76 MHz to 88 MHz, and 174 MHz to 216 MHz for fixed WSDs, and from 470 MHz to 698 MHz for fixed and portable WSDs. Finally, Canada reserved the same frequency ranges as the FCC, excluding the sub-band from 608 MHz to 614 MHz allocated for Radio Astronomy [25,27,28,30,31].

In Brazil, the Agência Nacional de Telecomunicações (Anatel) published a resolution that allocates frequency bands for the use of Idle Spectrum Devices, related to the TVWS regulations. The regulated frequencies resemble those defined by the FCC, excluding the bands from 76 MHz to 88 MHz and 608 MHz to 614 MHz. Another definition by Anatel is that initially, the maximum power of secondary users cannot exceed 1 W, or 30 dBm, peak, which may restrict the technology’s predictions for long-range applications. However, international studies suggest that greater flexibility in this parameter could significantly expand TVWS coverage without compromising DTV reception [32]. Requirements for limits on OOBE and spurious components, as well as additional technical conditions for operation, are yet to be established [33].

Among the defined parameters aimed at protecting primary users, it is noted that power limitation most directly affects current technologies in mobile communication systems, as most of these systems employ multicarrier waveforms [34]. Modulations such as OFDM, applied in current standards like long-term evolution (LTE) [35], LTE-Advanced (LTE-A) [36], and 5G new radio (5G NR) [37], for instance, were not conceived to operate in secondary networks. More recent techniques, such as filtered orthogonal frequency division multiplexing (F-OFDM) and generalized frequency division multiplexing (GFDM) may be appropriate to better meet the boundary conditions for operation in TVWS [38,39]. Particularly, low OOBE and the ability to control the peak-to-average power ratio (PAPR) are significant factors of interest for TVWS. The laboratory tests conducted in this paper can contribute to verifying if the regulation of using idle UHF channels for telecommunication services can ensure acceptable levels of adjacent channel leakage–power patio (ACLR) or absolute values of adjacent channel power emissions (ACPEs) while minimizing the impact of power restrictions on network coverage and capacity.

This paper presents the results of tests conducted within the scope of the project “Implementing TV White Spaces (TVWS) for Internet Access in Brazil: challenges and opportunities”, coordinated by the Brazilian Network Information Center or Núcleo de Informação e Coordenação do Ponto BR (NIC.br), and developed in collaboration with Instituto Nacional de Telecomunicações (Inatel) and the Universidade Federal do Ceará (UFC). The main objective is to evaluate the use of TVWS technology in Brazil and contribute to the process of regulation and standardization to establish an efficient and secure spectrum exploration method for broadcasters. Currently, one of the main regulatory challenges faced is the transmission power limitation of 1 Wp, which can significantly impact the feasibility of TVWS for long-range applications. Additionally, the need to ensure the harmonious coexistence of DTV and TVWS systems without mutual interference imposes technical constraints that must be experimentally evaluated. Thus, this study aims to bridge this gap through laboratory and field tests, providing concrete data to support future regulatory decisions.

The conducted studies present the conditions under which DTV and TVWS systems can coexist harmoniously, operating on adjacent channels without mutual interference. Additionally, they are expected to provide insights for establishing operational limits to make the effective implementation of TVWS networks viable in Brazil. Accordingly, this study investigates the impact of power limitation on TVWS system coverage, based on field tests conducted with the Remote Area Access Network for the Fifth Generation (5G-RANGE) system, and evaluates the necessary regulatory adjustments to ensure the viability of this technology without compromising DTV reception.

This study includes laboratory experiments related to the evaluation of the coexistence of TVWS signals with the DTV system adopted in Brazil. The DTV standard employed in the tests was integrated services digital broadcasting–terrestrial (ISDB-T) [40], coexisting with other signals such as fourth-generation (4G) cellular mobile networks and 5G, as well as the 5G-RANGE system [41,42,43,44], specifically developed for operation in the TVWS spectrum. From the laboratory experiments, the protection ratio (PR) values between the DTV signal and interfering signals were identified, ensuring no perceptible errors in the reception and demodulation of the DTV signal. These results are fundamental for establishing technical parameters that can be used in TVWS regulation in Brazil.

Additionally, field tests were conducted considering short and long distances, aiming to complement the evaluation of the impact of Resolution No. 747 of the Anatel [33], on the performance of this type of system and to confirm the results obtained in laboratory tests. Lastly, tests considering long distances assessed the range and throughput expected from the 5G-RANGE system when operating with 1 Watt of total peak power (Wp) delivered to the antennas, which refers to the power currently imposed on TVWS services. Long-distance tests were conducted considering Line-Of-Sight (LOS) communication to highlight the impact of transmit power limitation on maximum system coverage. This scenario enables a clearer understanding of the impact of power limitation without involving additional variables present in Non-Line-Of-Sight (NLOS) communication. The long-distance tests were conducted in a rural environment because this is the key environment considered for 5G-RANGE system conception and is the scenario considered in previous reference field tests [45].

The results obtained in this study provide concrete evidence of the challenges and opportunities of implementing TVWS in Brazil. The tests demonstrate that, while the power limitation imposed by current regulations significantly restricts the coverage of the technology, adjustments such as relaxing this restriction could enable its adoption without compromising DTV reception. Moreover, the technical analysis based on experimental measurements allows for the proposal of technical parameters that can be adopted to improve regulations and make TVWS implementation more efficient and accessible.

This paper is systematized as follows: Section 2 presents the conception, execution, and results of laboratory tests, defining the PRs to be observed. Section 3 outlines the methodology used for the execution and analysis of each field test related to the coexistence tests between DTV and TVWS. Section 4 showcases the results obtained in tests evaluating the impact of power restriction on the range and throughput in the 5G-RANGE system. Finally, Section 5 describes the main conclusions of this paper.

## 2. Proposal of the New Protection Definition

This section outlines the setup and procedures for the laboratory tests, along with the achieved results. The laboratory tests aim to establish the limit parameters of protection to ensure that TVWS systems do not interfere with the DTV system.

### 2.1. The Methodology and the Setup Used for the Laboratory Tests

The main laboratory test goal was to identify the PR limits of DTV receivers operating under the influence of interfering signals. The PR represents the minimum value of the signal-to-interference ratio at which a specific reception quality is achieved under specific conditions. The nomenclature used to display the test results regarding the occupied channel is that the signal under analysis, namely the DTV signal, occupies channel *n*, while the interfering signal occupies neighboring channels to the desired channel and is identified as, for example, n−1 and n+1, when interference is applied in the adjacent lower and upper channels, respectively. The interference applied in the second lower or upper channel is further identified as n−2 or n+2.

In the context of coexistence with the ISDB-T standard, it is possible to mention the ITU-R BT.1368−13 [16] recommendation as a baseline reference, which provides PR values for interference in the n−1 and n+1 channels as −26 dB and −29 dB, respectively. This relationship was defined for the ISDB-T system operating with a BW of 6 MHz, 64-QAM modulation, and a 7/8 encoding rate, and in the presence of interference from a signal also compliant with the ISDB-T standard. It is worth noting the PR values mentioned were defined for a bit error rate (BER) of $2\times 10^{-4}$ measured before the Reed–Solomon decoder.

The methodology adopted for conducting the laboratory tests involved evaluating the PR based on a subjective analysis of the decoded DTV video signal. This evaluation aimed to determine the threshold at which interference begins to degrade the quality of the received signal, using the quasi error free (QEF) and Threshold of Visibility (TOV) criteria.

The QEF condition was established as the benchmark for distinguishing between an acceptable and a degraded signal. A signal is considered to meet QEF criteria when its decoded video is free from visually perceptible distortions, artifacts, or freezing over a 60 s observation period. The TOV is determined by progressively increasing the interfering signal level until visible artifacts, errors, or interruptions appear in the decoded video. This marks the point at which interference starts affecting reception, providing a reference for identifying the initial stage of signal degradation.

Once the TOV threshold was identified, the interfering signal was gradually attenuated in 1 dB steps until the decoded video no longer exhibited any visible artifacts. At this point, the PR was calculated by [16,46]

(1) $$\begin{array}{c} PR\;\left(dB\right)=P_{\mathrm{desired}}-P_{\mathrm{interfering}}, \end{array}$$

where *P*_desired_ is the power of the desired signal in decibel-milliwatts (dBm) and *P*_interfering_ is the power of the interfering signal in dBm, at which the reception was restored to a QEF condition.

Thus, the recorded measurements reflect the subjective analysis of the decoded video signal under QEF conditions, ensuring that the PR values accurately represent the interference tolerance limits of the DTV system.

**Figure 1:**
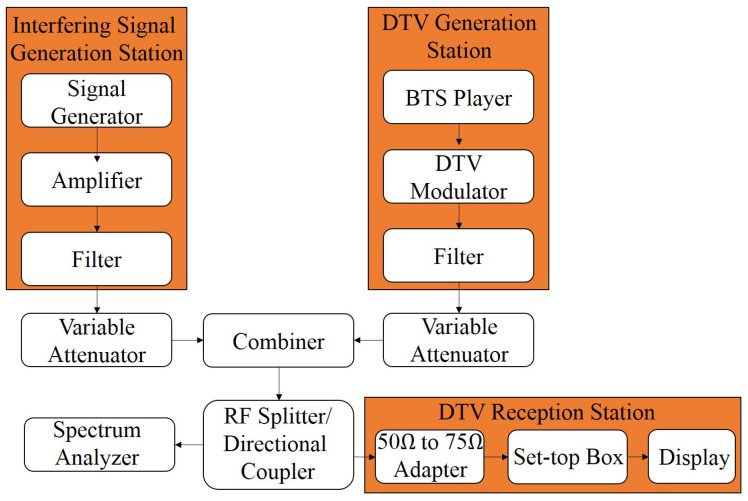
Block diagram of the test setup in the laboratory.

Figure 1 presents the setup diagram used to carry out the laboratory tests. The setup included a DTV generation station and another generation station of different technologies, in order to generate the called interfering signals. Additionally, the setup includes amplifiers, filters, attenuators, and radiofrequency (RF) combiners.

The DTV signal was generated using a broadcast transport stream (BTS) player, model digital video recorder generator (DVRG), manufactured by Rohde & Schwarz based in Munich, Germany, along with a professional DTV modulator, model E-Compact, manufactured by Hitachi Kokusai Linear based in Santa Rita do Sapucaí, Brazil. Channel filters, model 4352 from Hitachi Kokusai Linear, were configured on channels 46, 47, 48, 49, and 50 with 6 MHz of BW. The variable attenuators used were from HP based in Palo Alto, CA, USA, whose values and models varied depending on the desired configuration during the tests. The values used were 11, 12, 70, and 120 dB, and their models are 8494A, 355C, 8495A, and 355D, respectively.

The DTV modulator was configured to operate with the following parameters:

- Transmission channel n=48 UHF (674 MHz to 680 MHz);
- Channel BW: 5.572421 MHz;
- Parameters defined by the BTS flow:
  Transmission mode 3;
  Guard interval 1/8;
  Layer A—one segment, quadrature phase shift keying (QPSK) modulation, forward error correction (FEC) 2/3;
  Layer B—12 segments, 64 quadrature amplitude modulation (QAM), FEC 3/4.

The transmission power of the DTV system and the RF attenuator were adjusted so that the following powers were delivered to the tested set-top box (STB): −77 dBm, −70 dBm, −60 dBm, −50 dBm, −40 dBm, −30 dBm, and −20 dBm. It is noteworthy that the −77 dBm value represents the reception sensitivity threshold, and the −20 dBm value is the maximum recommended reception level according to the *Associação Brasileira de Normas Técnicas* (ABNT) Name-Based Routing (NBR) 15604 standard [47]. The other power levels were defined to obtain a wide range for the analysis of the behavior of the tested receivers.

The 5G-RANGE transceiver employing GFDM waveform for transmission [43] was considered as the interfering signal generator, representing a promising TVWS solution. The main characteristics of 5G-RANGE conception are summarized in Table 1 [48]. The 5G-RANGE conception offers support for five different numerologies, each one with specific number of subcarriers, subcarrier spacing, Cyclic Prefix (CP) duration, and Cyclic Suffix (CS) duration. Each numerology supports a different terminal velocity and offers support for multipath protection, based on CP duration, which impacts theoretical system coverage. The modulation supported are QPSK, 16-QAM, 64-QAM, and 256-QAM. For the field tests mentioned, the adopted numerology was numerology 0, which offers support for long-distance coverage. The system conception considers a connection with a remote TVWS database to define its initial operation frequency. The TVWS database suggests channels that are not licensed for TV broadcasters. Additionally, 5G-RANGE considers the usage of the spectrum sensing algorithm, which is periodically executed to analyze the spectrum and determine if the frequency of operation in usage is really free.

**Figure 2:**
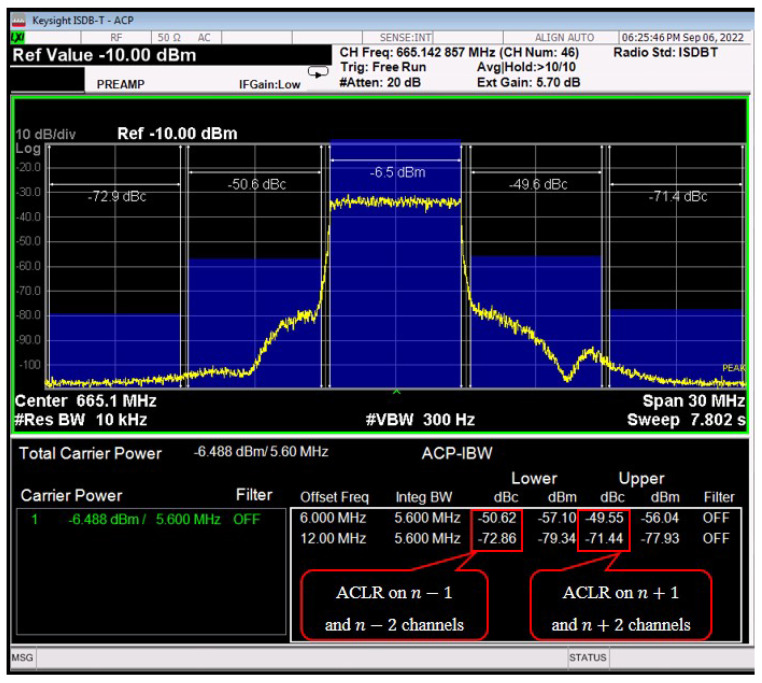
Spectrum of the 5G-RANGE using GFDM during the laboratory tests.

In addition to 5G-RANGE, a prototype eNodeB/gNodeB generator from Inatel was used for commercial 5G NR and 4G/LTE standards. It is important to mention that the signals for 4G and 5G technologies were generated using the same hardware but with different software configurations following distinct test modes and standards. A power amplifier from the manufacturer RFHIC based in Anyang, South Korea, model RWP05040-1H, with 36 dB gain and a channel filter, were employed for each interfering signal generator to represent the real operating conditions of these systems in the field. A variable attenuator was also used to facilitate the proper adjustment of the power level at the input of the DTV receiver during the tests. Table 2 presents the BW and power configurations of the generated interfering signals, along with their operating mode. The power value shown refers to the maximum level delivered to the input of STB. Additionally, Figure 2 presents the measurement of the generated and characterized 5G-RANGE signal for laboratory tests. The ACLR values on channels n−1 and n+1 are observed to be −50.62 dB and −49.55 dB, and the ACLR on channels n−2 and n+2 are −72.86 dB and −71.44 dB, respectively. ACLR is the ratio, in dB, between the transmitted power in the assigned channel and the transmitted power in the adjacent channel.

Given that, in the laboratory tests, the DTV modulator was configured to transmit on the UHF channel n=48, the interfering signal was alternately allocated in neighboring UHF channels 46, 47, 49, and 50, referred to as interference on channels n−2, n−1, n+1, and n+2, respectively.

The outputs of the variable attenuators of the DTV and interfering signal generation are combined to occupy the same medium and transmission spectrum. They are then divided by an RF splitter to allow simultaneous analysis of the combined signals. The first output of the RF splitter is connected to the input of the DTV reception station, where there is a 50 Ω-to-75 Ω impedance adapter, commercial DTV receivers (STBs), and the display for video visualization. STBs from three different manufacturers were used, employing Silicon Tuner technology, including two commercial ones and one fabricated and donated to families participating in the Brazilian government program called “*Bolsa Família*”. The second output of the RF splitter is connected to the spectrum analyzer, model MXA N9020A signal analyzer, manufactured by Agilent. The power level measurements of the DTV signal and the interfering signal were performed using the mentioned spectrum analyzer in adjacent channel power (ACP) mode, with a resolution bandwidth (RBW) of 10 kHz, a video bandwidth (VBW) of 300 Hz, and a span of 30 MHz, while the center frequency depended on the channel being analyzed.

### 2.2. Main Results Achieved

**Figure 3:**
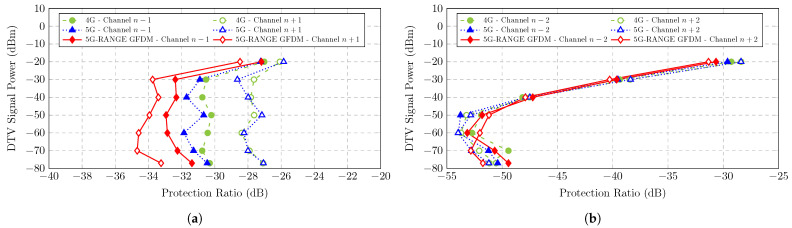
DTV power versus limit values of PR for interference signals generated by different technologies applied on channels: (**a**) n+1 or n−1. (**b**) n+2 or n−2.

The results obtained from the laboratory tests were condensed into several curves, which are presented and described below. Figure 3 depicts curves that relate the DTV signal power to the average PR obtained for the demodulation and decoding of the video signal without visually perceptible issues. Therefore, the PR value presented is the limit value for harmonious coexistence, according to the aforementioned criterion. Each curve on the graph represents the mean PR value, named as the PR_avg_ value, obtained for the three evaluated receivers. Each curves is related to the influence of one interfering signal at a time, generated by one of the three different systems described: 4G, 5G, or 5G-RANGE in GFDM.

Figure 3a illustrates the DTV signal power versus PR_avg_ curve for a non-simultaneous interfering signal applied to the immediately lower or upper adjacent channels, i.e., interference on channel n−1 or n+1. It can be observed that for the DTV signal with a power lower than −30 dBm, the average PR value for different technologies, considering the incidence of interference in one of the adjacent channels, is around −30 dB. In other words, the DTV signal can be correctly demodulated even in the presence of an interfering signal in an adjacent channel with a power around 30 dB higher than the DTV signal. However, for DTV power levels higher than −30 dBm, the PR value must be higher than the mentioned value, implying a lower power of the interfering signal. The highest PR_avg_ value identified was approximately −25.85 dB for the DTV power of −20 dBm when the interfering signal was the 5G NR signal on channel n+1. Additionally, it is observed that the lowest PR_avg_ values for interference on channels n−1 and n+1 are achieved with 5G-RANGE in GFDM as the interferer, highlighting its low OOBE.

Since the received power of the DTV signal in real conditions is typically reduced due to the distance between the receivers and transmission stations, it is worth noting the PR_avg_ values obtained for the DTV signal power of −77 dBm, which is the recommended sensitivity threshold for DTV receivers [47]. The lowest PR_avg_ values for interference on channels n−1 and n+1 are −31.38 dB and −33.24 dB, respectively, both achieved with 5G-RANGE in GFDM as the interfering signal. On the other hand, the highest PR_avg_ values were measured for the 4G/LTE and 5G NR technologies, which were −30.31 dB for channel interference n−1 and −27.07 dB for channel interference n+1.

Figure 3b shows the DTV signal power versus PR_avg_ curve for a non-simultaneous interfering signal applied to the second lower or upper adjacent channel, i.e., interference on channel n−2 or n+2. It can be observed that for the DTV signal with a power lower than −50 dBm, the average PR value for different technologies, considering the incidence of an interfering signal, is around −52.5 dB. In other words, the DTV signal can be correctly demodulated even in the presence of an interfering signal in the second adjacent channel with a power level around 52.5 dB higher than the DTV signal. However, the PR value must be higher than the mentioned value for DTV power levels higher than −50 dBm. The highest PR average identified was approximately −28.4 dB, for the DTV power of −20 dBm when the 5G NR and 4G/LTE signals were the interfering signals in the channel n+2.

Similarly to the analysis performed under interference conditions n−1 and n+1, for the received DTV power of −77 dBm, the lowest PR_avg_ values for interference on channels n−2 and n+2 were −51.16 dB and −51.74 dB, achieved with the 4G/LTE technology and **the 5G-RANGE** transceiver using GFDM, respectively. The highest values were also obtained with the same technologies, with 5G-RANGE as the interfering signal on channel n−2 with a PR_avg_ of −49.43 dB and 4G/LTE technology as the interfering signal on channel n+2 with a PR_avg_ of −50.54 dB.

Additionally, during the test campaign, the PR was investigated in scenarios where it had two neighboring channels simultaneously around the DTV channel. For this, there was an adjustment in the test setup, including another interfering signal generation station, and combining the outputs of the variable attenuators of each one to work with two interfering signals. In these tests, signals 4G and 5G-RANGE in GFDM were used on channels n−1 and n+1, respectively. Table 3 shows the results of laboratory tests considering the incidence of two interfering signals simultaneously on channels n−1 and n+1. A 3 dB increase in the PR value is observed compared to the incidence of only one of the interferences at a time.

## 3. TVWS-DTV Coexistence: Field Measurements

The objective of the field tests was to complement the laboratory results. These tests allowed the evaluation of the operational limits of DTV STBs in the presence of interfering signals in adjacent channels in an outdoor scenario with wireless transmission, unlike the laboratory tests, which exclusively used wired transmission. The field tests were divided into short distances and long distances.

### 3.1. Field Tests Considering Short Distances

Short-distance tests were conducted within the Inatel campus, implementing links with distances of 15 and 50 m between the interfering signal generation station and the reception setup. It is important to note that, in these field tests, only the 5G-RANGE transceiver employing the GFDM technique was used as the interfering signal.

The chosen distances between the interfering signal generation station and the mobile receiving unit allowed for achieving PRs representing the operational limits of DTV STBs. This allowed validation of the limit conditions found in laboratory tests. The tests provided a PR of around −30 dB for interference on channels n−1 and n+1 and a PR of around −50 dB for interference on channels n−2 and n+2.

Next, more details will be presented regarding the characterization of the test environment for short distances, the interfering signal, and the results obtained.

#### 3.1.1. Field Test Setup and Methodology Used

**Figure 4:**
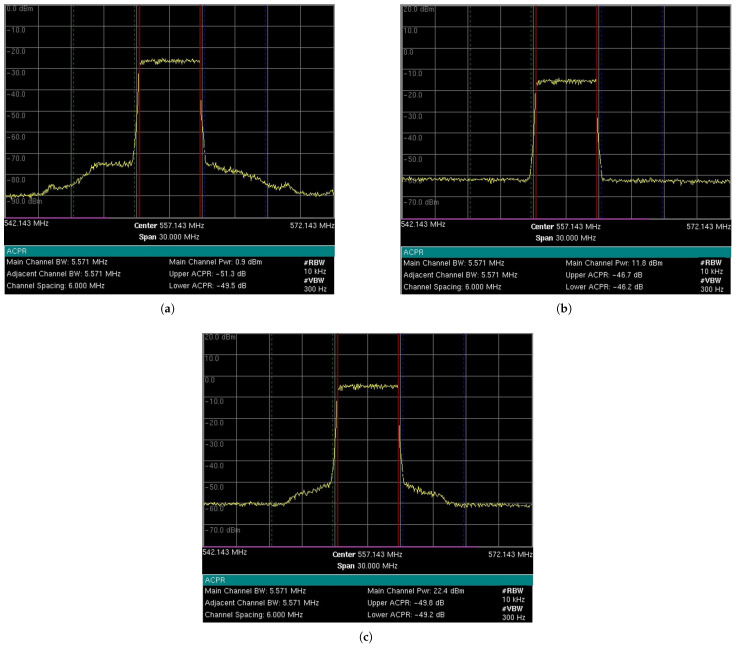
Spectrum of the GFDM signal transmitted by one of the antennas of the base station for a power of (**a**) 1 dBm, (**b**) 12 dBm, and (**c**) 22 dBm. The red vertical lines represent the bandwidth limits considered for the main channel, while the green and blue lines indicate the boundaries of the adjacent channels used for ACPR measurement.

The 5G-RANGE base station (BS) was configured to use the GFDM technique with a BW of 5.571 MHz, transmitting on channel 28 in the UHF band. Figure 4 shows measurements made by the spectrum analyzer, model MT8222B, manufactured by Anritsu, specifically the power measurement of the center channel at a frequency of 557.143 MHz with a bandwidth of 5.571 MHz, as well as the ACLR measurements for the adjacent channels. The graphs show the received signal power level and the frequency on the *y* and *x* axes, respectively. The measurement parameters include an RBW of 10 kHz, a VBW of 300 Hz, and a span of 30 MHz. Figure 4a shows the characterization of the RF signal delivered to one of the antennas for a transmission power of around 1 dBm root mean square (RMS) per antenna. As 5G-RANGE uses multiple-input and multiple-output (MIMO) 2 × 2, the total power transmitted by the BS is 4 dBm RMS or 2.51 mW RMS. Considering a PAPR margin of 10 dB, the total peak power transmitted (*P*_TX_total__ TVWS) in this condition is about 14 dBm or 0.03 Wp. The adjacent channel power ratio (ACPR), which, like the ACLR, establishes the relationship between the transmission channel power and adjacent channels, for the lower and upper channels in this condition is −49.5 dB and −51.3 dB, respectively. The short-distance tests, which allowed reaching the operational limit of DTV STBs, considered powers close to this description, i.e., with transmission around 1 dBm RMS per antenna. It is worth noting that the tests were conducted over short distances, where free-space propagation attenuation is significantly reduced compared to over long distances, allowing the achievement of the operational limit conditions of STBs with a lower interfering signal power. However, the main metric to be observed in the tests was the PR value on the link.

The short-distance tests, considering interference on channels n−2 and n+2, which allowed reaching the operational limit of DTV STBs, considered 5G-RANGE BS transmission powers close to 12 dBm and 22 dBm. Figure 4b shows the characterization of the RF signal delivered to one of the antennas for a transmission power of around 12 dBm RMS per antenna. The total power transmitted by the BS in this case is 15 dBm RMS or 0.03 W RMS. *P*_TX_total__ TVWS in this condition is 25 dBm, or 0.32 Wp. The ACPR for the lower and upper channels in this condition is −46.2 dB and −46.7 dB, respectively. Figure 4c presents the characterization of the RF signal delivered to one of the antennas for a power of around 22 dBm. The total power transmitted by the BS in this case is 25 dBm RMS or 0.32 W RMS. *P*_TX_total__ TVWS in this condition is 35 dBm, or 3.16 Wp. It is worth noting that this last power value considered for short-distance tests is higher than the 1 Wp limitation imposed by TVWS regulation in Brazil. The ACPR for the lower and upper channels in this condition is −49.2 dB and −49.8 dB, respectively.

**Figure 5:**
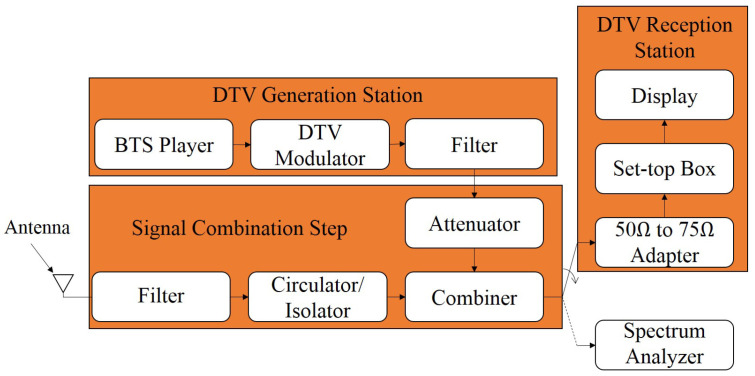
Block diagram of the MS setup in short-distance tests.

The transmission power values of 5G-RANGE that allowed reaching the operational thresholds of DTV STBs for each test condition are detailed in Section 3.1.2. Although some of the operational limit conditions of STBs were achieved with slightly different transmission powers of 5G-RANGE than those shown above, it is understood that the presented characterizations allowed for identifying the conditions of the interfering signal in each test. Regarding the reception station, Figure 5 illustrates the measurement setup that forms the mobile station (MS). The setup includes a BTS generator, model DTU-245 from Dektec based in Utrecht, The Netherlands, to provide a BTS stream in the DTV modulator input, which was generated with the same configuration used in the laboratory tests. Additionally, the MS consists of a Signal Combination Stage and a complementary arrangement for data collection and analysis.

The DTV modulator used is a professional equipment manufactured by Hitachi Kokusai Linear. The output signal of this modulator is delivered to a channel filter to eliminate traces of image frequency or other spurious signals that may compromise the measurements. After filtering, the signal passes through RF attenuators adjusted to provide power around −77 dBm at the input of the DTV STB (*P*_RX_ DTV) (after the impedance adapter from 50 Ω to 75 Ω), since this value corresponds to the minimum sensitivity recommended by the standard for DTV STBs. Thus, the proposed coexistence tests evaluate the coexistence of DTV with TVWS in the condition of receiving the DTV signal at the standardized sensitivity limit.

The TVWS signal generated by the BS is received via an antenna and is filtered to minimize the influence of other signals present in the channel. An RF isolator, model RFSL6105-01 from RFCI based in San Jose, CA, USA, was used to prevent the signal generated in the setup from being transmitted via the antenna. The TVWS signal is then combined with the DTV signal from an RF combiner, model 11667A, from Agilent based in Santa Clara, CA, USA. It is important to note that the combiner used attenuates the signals at its input by 6 dB. The resulting combined signal, called the setup output signal, is used in two ways: for test signal evaluation, using measurements collected with a spectrum analyzer, MT8222B manufactured by Anritsu based in Atsugi, Japan, in ACPR mode, with the RBW settings of 10 kHz, a VBW of 300 Hz, and a span of 33 MHz, while the center frequency depended on the channel being analyzed; and as the signal applied to the input of the DTV STB, after the correct impedance adaptation (from 50 Ω to 75 Ω).

All measurements taken on the spectrum analyzer take into account the insertion loss of the impedance adapter, which is 5.7 dB, to adequately display the signal actual levels applied at the input of the DTV STB. Three commercial DTV STBs from different manufacturers are used, which are the same equipment implemented in laboratory tests. The video output of the STBs was connected to a TV monitor, through which it is possible to visualize the video signal and, thus, conduct subjective analyses of the DTV signal quality.

The subjective analysis of video quality, referred to as DTV STB Status, runs for 60 s on each of the three STBs and is classified as follows:

- OK—video viewed without freezing or artifacts;
- Intermittent—video viewed, but with occasional freezing or artifacts;
- Not OK—no video playback or permanently frozen video.

**Figure 6:**
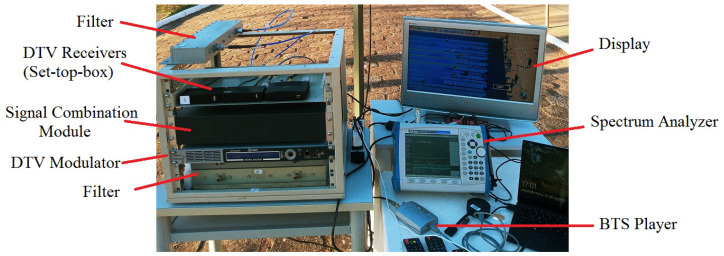
Photograph of MS setup used in short-distance tests.

In order to better illustrate the test conditions, Figure 6 presents a photograph of the setup described above, whose block diagram is shown in Figure 5.

In terms of channel allocation, tests were planned for DTV reception in the presence of interference applied to channels n+1, n−1, n+2, and n−2, where position *n* refers to the channel occupied by the desired signal, i.e., the DTV signal. The transmission frequency of the 5G-RANGE was set to UHF channel 28, while the DTV signal was adjusted to UHF channels 26, 27, 29, or 30, to allow tests with interference on channels n+2, n+1, n−1, and n−2, respectively. A filter was configured for each of these channels to properly tune the output signal of the DTV modulator. Table 4 summarizes the frequencies planned for the field test, the channel of application of the interfering signal for each condition, and the expected power to be applied at the input of the DTV STBs.

#### 3.1.2. Test Environment and Results

**Figure 7:**
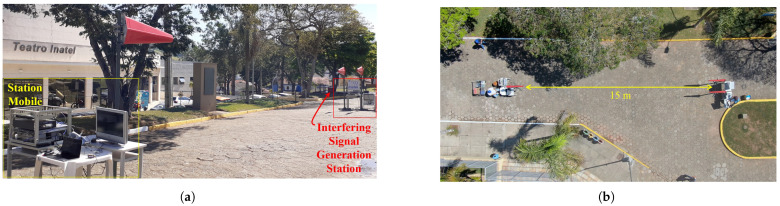
Test photographs: (**a**) Tests employing a distance of 15 m between the stations. (**b**) Aerial view of the test setup with a distance of 15 m between the stations.

**Figure 8:**
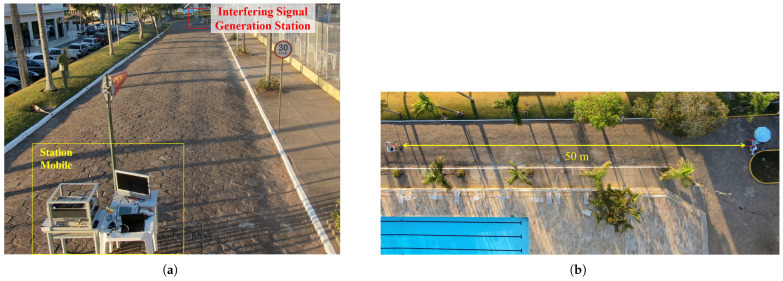
Test photographs: (**a**) Tests employing a distance of 50 m between the stations. (**b**) Aerial view of the test setup with a distance of 50 m between the stations.

The coexistence tests considering short distances were conducted within the premises of Inatel. For this purpose, a street on the campus near the Inatel Theater was selected, enabling the tests to be conducted with a direct line of sight and distances of up to 50 m between the stations. Figure 7a shows the test setup for a 15 m link distance. In the photograph, the interfering signal generation station is on the right, and the mobile or receiving station is on the left. In Figure 7b, there is an aerial view of the setup for the mentioned test condition. The estimated free-space attenuation for this distance is approximately 55 dB. Figure 8 shows the lateral and aerial views of the test setup with a 50 m distance between the stations. The free-space attenuation loss for this distance is approximately 66.3 dB.

The main objective established for short-distance tests was to evaluate the operational limit conditions of DTV STBs under the incidence of interfering signals in adjacent channels. Thus, the power of the interfering signal transmitter was adjusted to obtain PR values close to those identified in laboratory tests, given that the DTV signal reception power was adjusted to −77 dBm. Gradually, the transmission power of the TVWS signal (*P*_TX_ TVWS) was increased until the demodulated and decoded test video exhibited an “Intermittent” or “Not OK” DTV STB Status in the subjective analysis. Upon reaching this condition, *P*_TX_ TVWS was reduced until the video was viewed without freezing or noticeable artifacts, achieving an “OK” DTV STB Status. When this condition was reached, the test results were registered in tables, as presented below. Therefore, the data documented in the tables consider the limit value for the “OK” status reception condition for each STB. Additionally, the measurements of the DTV signal power (*P*_RX_ DTV) and the TVWS signal power (*P*_RX_ TVWS) delivered to the input of the DTV STB were recorded. Through these measurements, the PR value was calculated and registered. ACPR, which indicates the power relationship between the lower (“ACPR_Lower_”) and upper (“ACPR_Upper_”) adjacent channels and the DTV channel under test, was also measured.

As described previously, the PR and ACPR parameters play a fundamental role in determining the viability of TVWS technology in practical scenarios. PR defines the minimum level of protection required for the DTV signal to be received without noticeable degradation in the reproduced video, while ACPR reflects the unwanted spectral interference in adjacent channels. In rural areas, where the distances between the transmitting station and the receiver may be greater, the presence of high spectral interference may compromise DTV reception, even in locations where the signal level should be sufficient to guarantee quality of service. Therefore, the evaluation of these parameters is essential to determine operational limits that guarantee both the protection of DTV systems and the viability of TVWS services.

Table 5 presents the results of the measurements obtained for distances of 15 m and 50 m under the operational limit condition of each STB. The conditions of PR registered refer to the incidence of interfering signals on channels n+1 and n−1. The highest PR value recorded in this test was −32.7 dB for STBs 1 and 2 under interference on channel n+1 for the 50 m distance tests. On the other hand, the lowest recorded PR value was −35.5 dB, obtained with STB 3 under interference on channel n+1 and for a 15 m distance between the stations.

From the measured PR values, a PR_avg_ value can be calculated for the incidence of interfering signals on channels n+1 and n−1. The PR_avg_ value for interference on channel n+1 is −33.73 dB, while for channel interference n−1, it is approximately −34.32 dB. The difference between these values, about 0.65 dB, may be influenced by the fact that the interfering signal has a lower ACPR value in the upper adjacent channel.

Table 6 presents the results obtained under the conditions of interference applied to channels n+2 and n−2 for distances of 15 m and 50 m between the stations. In these tests, an inferior performance of STB 1 can be observed, with PR values approximately 10 dB higher than the other two STBs under the operational limit condition. The PR values for STB 1 under interference on channels n+2 and n−2 were around −46.05 dB and −45.1 dB, respectively, considering that these values represent the average between the PR values obtained for 15 m and 50 m. On the other hand, STBs 2 and 3 showed lower PR values, indicating better performance as they support an interfering signal power about 10 times higher than the interfering signal power supported by STB 1 under interference in the conditions of channels n+2 and n−2. Under the interference n+2 condition, the measured PR values for STBs 2 and 3 were around −55.08 dB, while under the interference n−2 condition, the values were approximately −55.15 dB. Similarly to the calculation performed under interference on channels n+1 and n−1, the PR_avg_ between the three STBs can be obtained. The PR_avg_ for the three STBs obtained under interference applied to channel n+2 was −52.06 dB, and for the interference applied to channel n−2, a value of −51.8 dB was calculated.

The coexistence tests demonstrated that the recorded PR values were close to and consistent with those obtained in the laboratory tests. Taking as an example the PR measured on channel n−1 and n+1 using 5G-RANGE as the TVWS signal, the values of −31.38 and −33.24 were obtained in the laboratory, while in the field tests, the values obtained were −34.32 and −33.73, respectively. Thus, when the PR was below the identified limit, the reception of the DTV signal was noticeably affected through video observation. Among the main effects observed, the presence of visual artifacts, video freezing, and intermittent failures in signal demodulation stands out. These results indicate that the appropriate definition of the PR values is essential to ensure that TVWS can operate without compromising the DTV user experience.

### 3.2. Field Tests Considering Long Distances

The long-distance field tests were conducted to collect information to complement the coexistence analyses of TVWS services with DTV systems, as well as to estimate the impact of the current power limitation on the maximum coverage of TVWS systems [33]. This section provides details about the methodology, setup, and key results obtained from the execution of the field tests. First of all, it is worth noting that several challenges had to be overcome in order to carry out the field tests. Among these, we can highlight the definition of the measurements performed, the logistics involved in installing the BS in a location with good coverage and preparing a mobile station, evaluating system coverage, and defining strategic locations for conducting field measurements. These challenges were addressed through the establishment of a clear methodology for conducting the tests, the generous provision of space in a shelter for BS installation, and the use of a simulation tool for coverage testing and selecting the measurement locations in the field.

#### 3.2.1. Base Station and the Mobile Unit Setup

**Figure 9:**
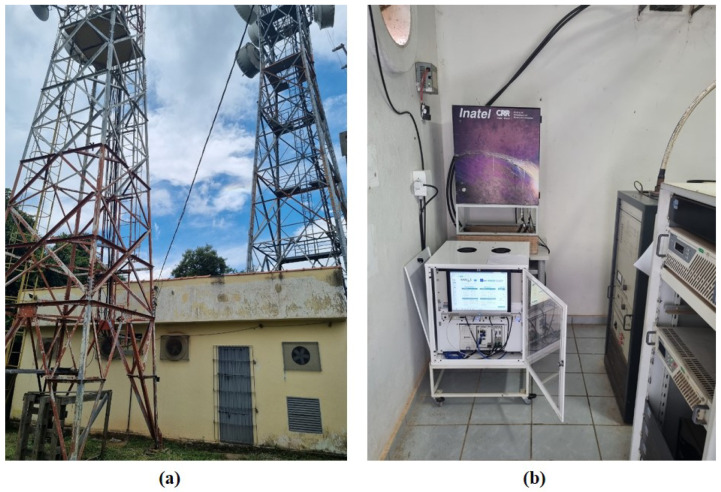
BS installed in *Serra do Paredão*. (**a**) Shelter selected for installation. (**b**) 5G-RANGE BS installed and activated.

A TVWS BS was strategically installed, and an MS was constructed to facilitate the measurement of the TVWS link’s quality and its coexistence with DTV in different locations. The TVWS system used in the tests was the 5G-RANGE transceiver [43]. The installation site for the TVWS BS was the *Serra do Paredão*, also known as “*Morro das* 3 *Torres*”, on the border of the cities of Santa Rita do Sapucaí and São Sebastião da Bela Vista, both in the state of Minas Gerais. The location features a masonry shelter and a tower where the antennas were installed. Figure 9 presents photos of the shelter and tower on the left (Figure 9a), as well as the 5G-RANGE BS installed inside the shelter on the right (Figure 9b).

**Figure 10:**
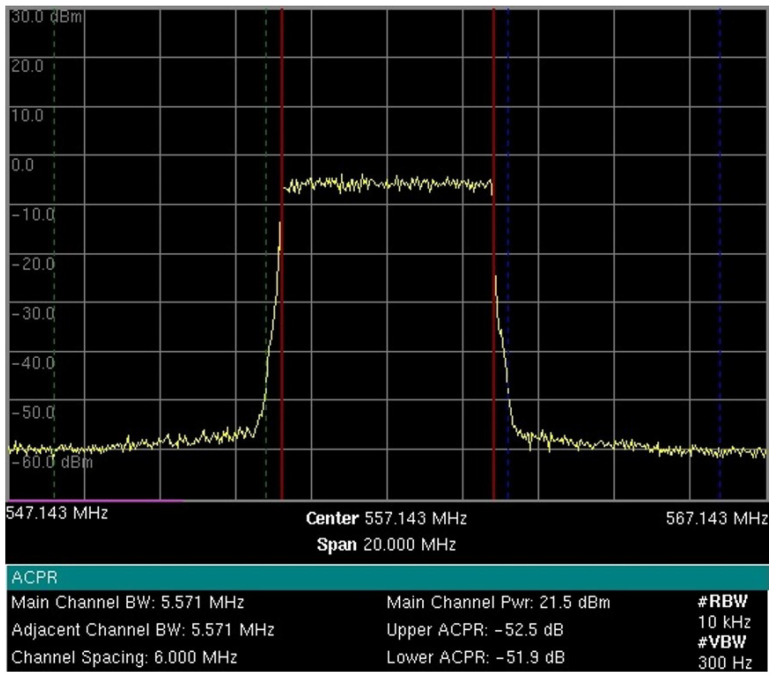
Spectrum of the GFDM signal transmitted by one of the antennas of the BS.

The 5G-RANGE BS was again configured to employ GFDM with a BW of 5.571 MHz on channel 28 of the UHF band and with an initial power of 1 Wp in total (*P*_TX_ TVWS). As 5G-RANGE employs a MIMO 2x2 technique, each antenna must transmit half of the total power allowed by the current regulation. Figure 10 shows the RF signal delivered to one of the BS transmission antennas.

**Figure 11:**
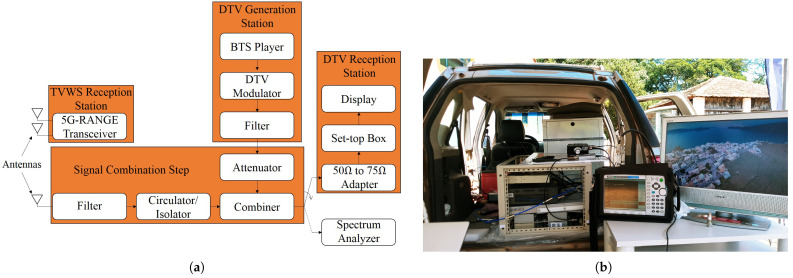
(**a**) Block diagram of the MS setup for long-distance tests. (**b**) MS installed in a vehicle.

Additionally, an MS is proposed to be installed in different locations. Figure 11a illustrates the block diagram that constitutes the MS. The setup includes a station for receiving the TVWS signal to investigate the communication link conditions of the 5G-RANGE system. This is the only block added compared to the setup presented for tests considering short distances. In other words, in addition to the TVWS Reception Station, the MS consists of a DTV Generation Station, consisting of a BTS player to generate the same BTS stream used in laboratory tests at the DTV modulator input, a Signal Combination Stage, a DTV Reception Station, and an isolated Spectrum Analyzer block.

Therefore, all preparations for data collection for the coexistence tests of DTV and TVWS were performed in the same way as the tests considering short distances. In other words, the DTV signal passes through a filter, attenuator, and combiner and is adjusted to provide power around −77 dBm at the input of the DTV STB (*P*_RX_ DTV), after the impedance adapter of 50 Ω to 75 Ω. The TVWS signal generated by the BS is received via an antenna, filtered, and combined with the DTV signal from an RF combiner. Finally, the combined signal is operated in two ways: to evaluate the test signal, based on measurements using a spectrum analyzer, and as a signal applied to the input of the DTV STB for visualizing and assessing the video signal.

Furthermore, all measurements made on the spectrum analyzer took into account the insertion loss of the impedance adapter, which is 5.7 dB. The three different DTV STBs were used again, and the subjective video quality analysis, referred to during the tests as DTV STB Status, was carried out in the same way as the short-distance tests, following the same classification mentioned earlier. Figure 11b shows the MS installed in a car.

Regarding channel allocation, for the long-distance field tests, reception tests of DTV were planned under the incidence of interference applied to channels: n+1, n−1, and n−2, where the position *n* refers to the channel occupied by the desired signal, which, in this case, is the DTV signal. In the laboratory tests, the DTV signal was fixed on one channel, and the interfering signals were switched to adjacent channels. On the other hand, in the field tests, the transmission frequency of 5G-RANGE, i.e., the interfering signal, was fixed to a specific channel for the field tests, which was UHF channel 28. This decision was made because the BS was located at considerable distances from the MS, making it impractical to go to the installation site of the interfering signal transmitter and modify its channel whenever there was a change in the interference condition analysis, since channel modification involves physically swapping some circuits of the transmitter. Thus, the DTV signal was adjusted to UHF channels 27, 29, or 30 to allow tests with interference on channels n+1, n−1, and n−2, respectively. A filter was configured for each of these channels to properly filter the output signal of the DTV modulator. Despite the need to physically change the circuit of the DTV signal generation, this part of the test setup was installed in the MS, making manipulation more feasible. It is worth noting that UHF channel 26 was not used due to the availability of components needed for the construction of the TVWS Reception Station. Additionally, a request for radiofrequency spectrum licenses was made for the long-distance tests, and the license for channel 26 was not obtained.

The received TVWS signal power (*P*_RX_ TVWS) depended on the location where the measurement was taken and the pointing of the antenna, which was fixed in a single direction for all tests. The attenuation loss related to the elements of the test setup, such as cables, adapters, filters, and RF combiner, was on the order of 15 dB. Furthermore, a low-noise amplifier (LNA) with a gain of approximately 22 dB to amplify the received signal was implemented after the reception antenna. The use of the LNA allowed for creating a second reception condition in each location, due to the practicality and simplicity of switching its state from on to off and vice versa. On the other hand, it can be understood that the gain of the LNA compensates for and exceeds the inherent attenuations in the setup of the MS.

#### 3.2.2. Transmission Power of the TVWS BS and Definition of Locations

In accordance with current regulations, it is established that the BS TVWS primarily operates with a power of 1 Wp (30 dBm) per 6 MHz channel [33]. The total average power in the transmission channel, in this configuration, is 20 dBm, considering the typical PAPR of the GFDM signal in the configurations used by the 5G-RANGE BS is 10 dB. In addition to the power of 1 Wp, power amplifiers were linearized for powers of 2 Wp and 4 Wp. These different powers were defined to expand the conditions of the interfering signal power in the DTV system and to allow the evaluation of the impact of transmission power on coverage and TVWS service quality.

The linearization is crucial because increasing the transmission power implies an increase in the OOBE, and consequently, an increase in the values of ACLR or ACPR ratios. These ratios establish the relationship between the transmission channel power and the power of adjacent channels. If the OOBE level is above acceptable levels, techniques such as Digital Pre-Distortion (DPD) are employed, which requires parameterization and adjustment based on the operating frequency, transmission power, and characteristics of the amplifier used in each antenna. Table 7 shows the power delivered to each transmission antenna of the BS for each of the mentioned configurations. Unlike changing channels, adjusting the power in the BS can be achieved through remote access to the equipment, made possible by installing a network between Inatel and *Serra do Paredão* using a pair of radios that operate Wi-Fi in the 5 GHz range. The Inatel internal network was accessed through a Virtual Private Network (VPN) via a mobile device.

From the definition of the average transmission power of the 5G-RANGE system, coverage predictions were made using the Radio Mobile software to define candidate locations for field measurements [49]. The 1 Wp condition was considered for coverage prediction, with the BS positioned in *Serra do Paredão*, whose latitude and longitude are −22.19764 and −45.74194, respectively, at an altitude of 1.385 m. A directional antenna with a gain of 9.5 dBd was positioned at a height of 12 m from the ground, pointing towards the Vintém neighborhood in the rural area of the city of Santa Rita do Sapucaí. The coverage prediction performed using the Radio Mobile tool was used as a starting point for defining candidate locations for field measurements. The coverage prediction made did not consider the terrain morphology and practical issues such as the actual direction of the transmission antenna and the occurrence of multipaths and reflections, among other phenomena, which can lead to discrepancies between predicted and measured values in the field. Therefore, the definition of locations for field measurements met the following criteria:

- Prediction of a minimum reception level of the 5G-RANGE signal of −80 dBm;
- Accessibility of the location;
- Availability of a safe area for parking the MS and installing reception antennas;
- Absence of obstructions for LOS communication with the BS;
- Availability of a cellular network signal to enable remote access to the BS and facilitate parameter changes in the system;
- Selection of at least one location that allows identifying the coverage limit of the 5G-RANGE system;
- Selection of at least one location that allows establishing communication links with a throughput close to 100 Mbps for the 5G-RANGE system.

**Figure 12:**
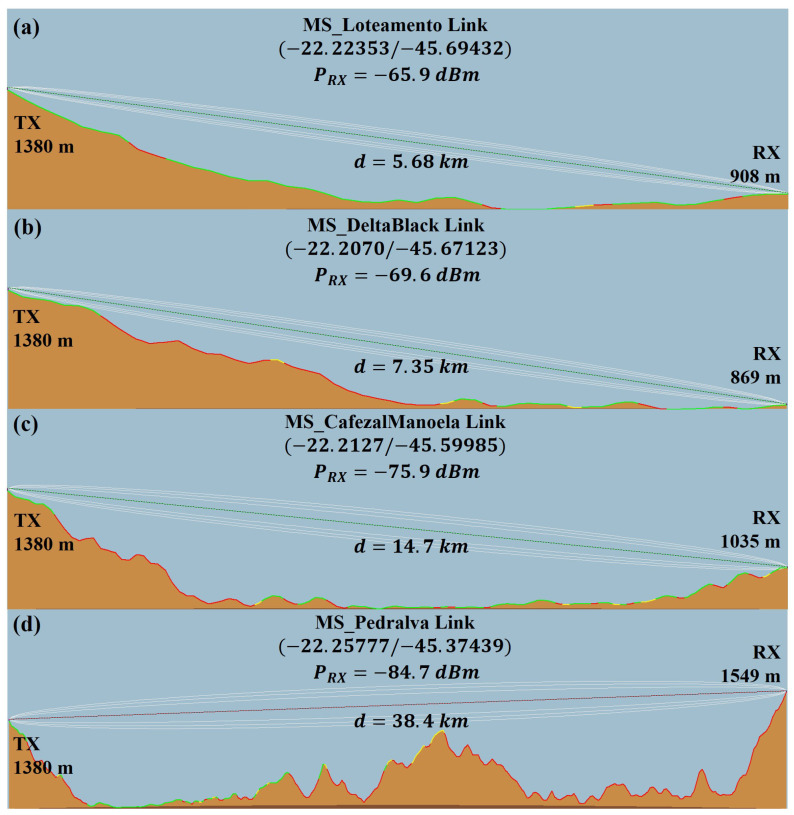
Relief profile and link information obtained by simulation for each MS position. (**a**) MS_Loteamento. (**b**) MS_DeltaBlack. (**c**) MS_CafezalManoela. (**d**) MS_Pedralva. The colored lines along the terrain represent signal power as a function of distance, where green indicates higher power levels, yellow represents intermediate levels, and red corresponds to the lowest power levels.

Figure 12 presents the chosen locations, as well as prediction information for each location and the topographic relief profile of the link. The chosen locations were named “MS_Loteamento”, “MS_DeltaBlack”, “MS_CafezalManoela”, and “MS_Pedralva”, which were located at straight-line distances (*d*) of 5.68, 7.35, 14.7, and 38.4 km from the BS, respectively. The selected locations, described below, cover a variety of environments typical of the interior of the country, allowing the analysis of TVWS performance under different propagation and use conditions:

- MS_Loteamento, located in a neighborhood far from the urban center, was chosen to represent expanding rural communities that, although far from central areas, have a higher concentration of residences and small properties. The objective of this location was to verify how TVWS can complement existing networks or act as a primary solution in regions with limited fiber optic or mobile network infrastructure;
- Delta Black Farm (MS_DeltaBlack) was selected because it represents large agricultural areas, where connectivity plays a crucial role in precision agriculture applications, remote monitoring, and automation of agricultural equipment. The lack of fixed telecommunications infrastructure in the region reflects the challenges faced by rural producers, who depend on wireless solutions to modernize their operations. In addition, the topography of the location influences signal propagation, making this environment ideal for evaluating the effectiveness of TVWS in open agricultural lands.
- MS_CafezalManoela, located in Serra da Manoela, was selected to represent mountainous areas with agricultural production, a common scenario in several regions of Brazil. The choice of this location also reinforces the importance of TVWS in connecting isolated rural communities, which often depend on agriculture and livestock farming and have low population densities. This specific point was chosen because, despite the high relief, it still maintains LOS with the base station, allowing the evaluation of TVWS signal propagation in a high-altitude environment, but without direct obstructions.
- Finally, MS_Pedralva, located in an olive grove on top of a mountain between the cities of Pedralva-MG and Maria da Fé-MG, was chosen to test the limits of TVWS system coverage, as it is located 38.4 km from the base station, in high and rugged terrain. This environment represents extreme scenarios of rural connectivity, where telecommunications infrastructure is practically non-existent and communication depends exclusively on long-range wireless solutions. The choice of this location also allowed us to assess the impact of signal attenuation due to the terrain, verifying the viability of TVWS in mountainous communities, a challenge present in several regions of Brazil.

#### 3.2.3. Presence of Interfering Signal in the Licensed Frequency Range for the Test

As previously mentioned, conducting field tests considering long distances involved requesting a *serviço especial para fins científicos e experimentais* (SEFICE) license, which is the Specialized Radiofrequency Station Installation License, from Anatel [43]. The license requested by Inatel considered four UHF channels for downlink and four channels for uplink, totaling a maximum width of 24 MHz per link. The frequency band planned for the transmission of the fixed TVWS station comprised the UHF channels 27, 28, 29, and 30. These channels were available according to the documents on the Anatel website.

It is important to highlight that during the field tests, a DTV signal was identified occupying the UHF channel 30, which was not licensed in the Anatel system. The presence of the DTV signal, considered as “interfering” for the tests, was mitigated by using a bandpass filter at the reception side, referred to as Filter_RX_, configured to receive channel 28, which is the transmission channel of the fixed TVWS station. However, the undesirable signal was also used in some additional tests, as it represents a real field situation and allows exploring other test conditions and scenarios.

Although the use of the unlicensed DTV signal facilitated measurements, serving as a second interfering signal in some tests, the presence of this signal on a frequency that was supposed to be free highlights the importance of employing systems for electromagnetic spectrum monitoring. This function aims to complement the database information related to licensed primary signals and available frequencies to ensure the proper operation and harmonious coexistence of TVWS and DTV systems.

#### 3.2.4. Results from the First Stage of Field Tests

The first stage of the field tests includes measuring and analyzing the coexistence of the DTV system with a TVWS system, and the second stage, addressed in Section 4, investigates the impact of the transmission power limitation applicable to TVWS systems on the coverage and quality of the secondary network link, which implemented the 5G-RANGE system. The results of the first stage were divided into initial tests and complementary tests, as presented below:

- Initial test results for the coexistence of DTV and TVWS

The proposed coexistence tests of DTV with the TVWS system consider the generation of the DTV signal on up to three distinct channels (DTV Channel), aiming to evaluate the demodulation and decoding of this signal in the presence of the TVWS signal (5G-RANGE in GFDM) as an interfering channel. Six initial test conditions were established, considering the TVWS system configured to deliver the total powers (*P*_TX_ TVWS) of 1 Wp, 2 Wp, and 4 Wp to the transmission antennas at the transmission side, while on the reception side, the LNA_RX_ was deactivated and activated for each of the configured *P*_TX_ TVWS.

Additionally, the field measurements included the power of the DTV signal (*P*_RX_ DTV) and the TVWS signal (*P*_RX_ TVWS) delivered to the input of the DTV STB. Through these measurements, an important parameter to be evaluated in the coexistence analysis was calculated, which refers to the power ratio between the signal of the analyzed channel (DTV) and the interfering channel (TVWS), i.e., the PR. The ACPR was also measured, indicating the power ratio between the lower adjacent (“ACPR_Lower_”) or upper adjacent (“ACPR_Upper_”) channels and the DTV channel under test. The values of the parameters achieved through measurements were obtained by the MT8222B spectrum analyzer manufactured by Anritsu. In addition to the measurements, a subjective analysis of reception was conducted by observing the DTV STB Status for each of the three commercial DTV STBs for 60 s.

The power of the undesirable DTV signal, i.e., the interfering signal present on channel 30 (*P*_RX_ INT. (CH30)), was also monitored. The test with interference present on channel n−2 was executed only at the MS_CafezalManoela location, where the *P*_RX_ INT. (CH30) level was lower than −90 dBm, since for this test, the DTV signal should occupy channel 30. At the MS_Loteamento and MS_DeltaBlack locations, the level of *P*_RX_ INT. (CH30) was higher than −90 dBm, and the co-channel interference caused the decoding of the video signal to be impossible.

It is worth mentioning that coexistence tests of DTV and TVWS were not conducted at the MS_Pedralva location, located 38.39 km from the BS, because the TVWS signal level *P*_RX_ TVWS at that location was significantly reduced (−83.9 dBm for a *P*_TX_ TVWS of 1 Wp), causing no significant interference with the DTV signal.

The results of the coexistence tests considering *P*_TX_ TVWS up to 4 Wp, which is a power level 6 dB higher than the currently prescribed limitation for TVWS systems, did not cause harmful interference to the DTV signals in the executed tests. The condition with the lowest PR value tested for interference on channels n−1 and n+1 was −28.3 dB and −27 dB, respectively, at the MS_Loteamento location, which is closest to the 5G-RANGE BS with a distance of 5.68 km, and in these conditions, all STBs were able to demodulate the DTV signal normally. On the other hand, the condition with the lowest PR value tested for interference on channel n−2 was −16 dB at the MS_CafezalManoela location.

Table 8 presents the collected measurements for the MS_Loteamento location with LNA off and on. It can be observed that even though it is the closest location to the BS where the tests were conducted, the DTV signal did not suffer interference, even for the conditions of higher *P*_TX_ TVWS and LNA_RX_ on, which allowed amplifying the received interfering signal from the antenna by approximately 23 dB.

- Additional test results for the coexistence of DTV and TVWS

The complementary tests were executed to reach the operational limits of DTV and TVWS coexistence, since the initially proposed parameters did not allow achieving “Intermittent“ or “Not OK” DTV STB Status. Therefore, analyzing only the location closest to the BS (MS_Loteamento), the transmission power of the 5G-RANGE system was increased above 4 Wp. Tests were conducted with *P*_TX_ TVWS up to 10 Wp delivered to the transmission antennas. Expanding to powers above this value, an increase in OOBE was observed, and due to these new values of *P*_TX_ TVWS not being previously linearized, it would not be possible to conclude whether the reason for incorrect demodulation in the STBs was due to the protection ratio reaching the limit or the lack of linearization to reduce OOBE.

Under these power conditions and additionally considering the reception LNA in the on state, it was possible to obtain a PR of −30.2 dB, resulting in “OK” DTV STB Status for all three STBs after the MS’s setup. However, with lower PR values, it was possible to obtain “Not OK” or “Intermittent” DTV STB Status in two situations, as evidenced in Table 9. It is noteworthy that for a *P*_TX_ TVWS of 6.3 Wp, a PR of −30.2 dB was obtained, for which all three STBs had “OK” DTV STB Status. When *P*_TX_ TVWS was increased to 8 Wp, a PR of −31.1 dB was calculated, and STB 1 presented an “Intermittent” DTV STB Status. Finally, with a *P*_TX_ TVWS of 10 Wp, a PR of −31.9 dB was obtained, and STBs 1 and 2 had “Not OK” and “Intermittent” DTV STB Status, respectively.

**Figure 13:**
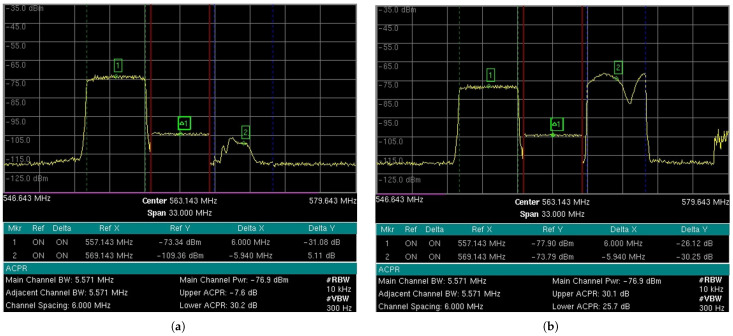
(**a**) Input spectrum of the DTV STB for 6.3 Wp and with LNA in reception. (**b**) Input spectrum of the DTV STB for 4 Wp, with LNA in reception, removing the filter, and with a 4 dB attenuation in reception.

The input spectrum of the DTV STBs in the threshold condition for all three STBs to work simultaneously is shown in Figure 13a. The DTV signal is in the center of the spectrum, and the TVWS signal is on the left, while the signal on the right is the vestige of the interfering DTV signal present on channel 30 after filtering.

Additionally, at the MS_Loteamento location, tests were conducted considering the simultaneous interference on channels n+1 and n−1. In these tests, the DTV signal under analysis was configured on channel 29, and the *P*_TX_ TVWS was set to 4 Wp, considering the LNA_RX_ on and without using Filter_RX_. This allowed using the unlicensed DTV signal present on channel 30 as interference on channel n+1. In this configuration, *P*_RX_ TVWS was −47.2 dBm, and *P*_RX_ INT. was −43.1 dBm, with PR_(n−1)_ and PR_(n+1)_ calculated at −29.7 dB and −33.8 dB, respectively, and “Not OK” DTV STB Status were obtained for the three STBs under test.

Subsequently, attenuators were inserted after the LNA_RX_ to reduce the received power of the interfering signals. As shown in Table 10, in the condition with a PR_(n−1)_ value of −25.7 dB and PR_(n+1)_ value of −30.1 dB simultaneously, which was achieved with an attenuation of 4 dB, all STBs had “OK” DTV STB Status. Note also conditions with 3 dB attenuation, for which only STB 1 had a “Not OK” DTV STB Status, and 2 dB attenuation, in which only STB 3 had an “OK” DTV STB Status.

The input spectrum of the DTV STBs in the threshold condition for all three STBs to work simultaneously is shown in Figure 13b. The DTV signal is in the center of the spectrum, the TVWS signal is to its left (interference n−1), and to the right is the interfering DTV signal (interference n+1).

## 4. Power Restriction Impact in the 5G-RANGE as TVWS System

The results presented in this section refer to the second stage of field tests, considering long distances, and investigate the impact of the transmission power limitation applicable to TVWS systems on coverage and link quality. The secondary network is the 5G-RANGE system, and the tests consider a total transmission power (*P*_TX_) restriction of 1 Wp delivered to the transmission antenna, for a transmission channel with a 6 MHz BW, based on Anatel’s resolution [33].

As a key reference parameter for analyzing the impact of power limitation on 5G-RANGE coverage, previous field tests were conducted when no power limitation was defined, as detailed in [45]. The channel BW used in previous and reference tests was 24 MHz, and the *P*_TX_, considering a PAPR of 10 dB, was approximately 50.8 dBm or 120.2 Wp. With these configurations, the system operated with a throughput of 102 Mbps over a link distance of 50 km. It is worth noting that this was not the maximum coverage limit of the system under the described conditions. In the reference test, the goal was to verify long-range coverage while maintaining support for a throughput of 100 Mbps.

Table 11 presents the results of field tests related to the 5G-RANGE system operating under the current restrictions imposed on TVWS systems. The measurements were performed at locations closest to the BS, specifically at MS_Loteamento and MS_DeltaBlack, with straight-line distances from the MS of 5.68 km and 7.35 km, respectively. In both links, it was possible to establish reception with a modulation and coding scheme (MCS) configuration that provides the maximum data throughput supported by the system. This configuration involves using the 256-QAM mapping scheme and employs Polar FEC encoding with a coding rate of 5/6. Consequently, a bitrate of 30 Mbps was achieved for a channel with a BW of 6 MHz.

It is important to emphasize that the criterion used for selecting the configured MCS at each location is related to achieving a BER of 0, measured and observed within a time interval of 5 s after resetting the system. Extrapolating to a transmission using four UHF channels, totaling a BW of 24 MHz, which is the maximum BW supported by the 5G-RANGE system, the MCS provides support for communication with a bitrate_4 Ch_ of 120 Mbps. Additionally, in Table 11, the received power (*P*_RX Ant._) captured by one of the system’s antennas and the signal-to-noise ratio (SNR) values are observed.

The results of the field measurements at the location referred to as “MS_CafezalManoela”, establishing a communication link with a distance of 14.71 km to the BS, are presented in Table 12. Tests were conducted at this location considering the BS configured to transmit, in addition to the initial *P*_TX_ of 1 Wp, power levels of 2 Wp and 4 Wp, since the initial *P*_TX_ did not provide the system with full operational capacity. However, the 1 Wp *P*_TX_ enabled the system to operate with an MCS featuring 256-QAM mapping and Polar code 2/3, providing support for a bitrate of 24 Mbps for the system operating with a 6 MHz BW. Considering a BW of 24 MHz, the bitrate_4 Ch_ of the system amounts to 96 Mbps, a value close to that obtained in the reference test of 5G-RANGE. Therefore, it is estimated that the maximum distance for the system to achieve a throughput of approximately 100 Mbps, for a 1 Wp *P*_TX_, is 14.7 km. Increasing the transmission power to 2 Wp and 4 Wp allowed the system to operate at the “MS_CafezalManoela” location with the MCS providing the highest throughput in the system, namely, 120 Mbps.

Table 13 presents the results of the tests conducted at the most distant location, which is at a straight-line distance of 38.34 km from the BS, referred to as “MS_Pedralva”. At this location, it was possible to establish a reception link using QPSK mapping and Polar code with a 1/2 rate in the 5G-RANGE system, for a *P*_TX_ of 1 Wp. This configuration supports a throughput of 4 Mbps for a transmission channel with a 6 MHz BW or 16 Mbps for a channel with a 24 MHz BW. Additionally, since this configuration provides the lowest MCS and the minimum throughput supported by the 5G-RANGE system, it can be concluded that, at the “MS_Pedralva” location, the system operated at the coverage edge for the configured 1 Wp *P*_TX_, which is the currently applicable transmission power limit for TVWS systems.

Tests were executed by increasing the total transmission power to 2 Wp, 4 Wp, and additionally, 8 Wp. Under these conditions, it was possible to achieve a communication link supporting a throughput_4 Ch_ of 28 Mbps, 48 Mbps, and 52 Mbps, respectively. This represents an increase equivalent to 1.75 times, 3 times, and 3.25 times, respectively, compared to the throughput supported with 1 Wp. The results of these tests showed that, for applications in rural areas, the power limitation imposed on TVWS may restrict coverage, which reinforces the need for regulatory adjustments that consider not only the protection of DTV, but also the viability of TVWS for long-distance communication.

**Figure 14:**
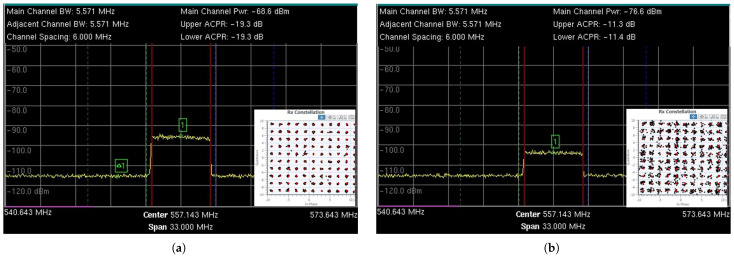

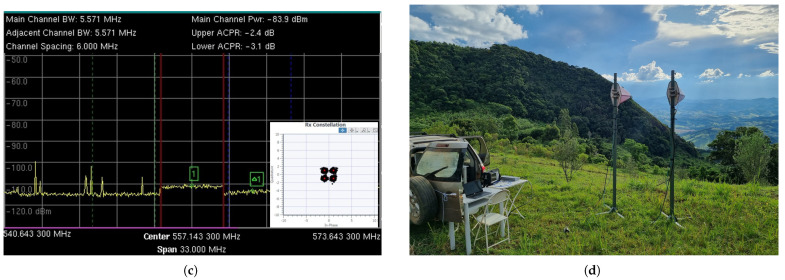
Reception spectrum and constellation of 5G-RANGE for the BS configured for 1 Wp at the locations: (**a**) MS_DeltaBlack. (**b**) MS_CafezalManoela. (**c**) MS_Pedralva. (**d**) Mobile station in MS_Pedralva during the field tests.

Figure 14d shows the MS at the “MS_Pedralva” location during the field tests, and Figure 14a–c illustrates the reception spectrum at the locations “MS_DeltaBlack”, “MS_CafezalManoela”, and “MS_Pedralva” for the BS operating with a power of 1 Wp. The *P*_RX Ant._ values are shown with a 6 MHz BW of the received signal. Additionally, there is an inset of the constellation corresponding to the modulation configuration achieved for each location.

## 5. Conclusions

This paper, inserted in the context of the project “Implementing TV White Spaces for Internet Access in Brazil”, provided essential information to support the implementation of TVWS technology in Brazil. This included evaluating the harmonious coexistence between DTV and TVWS systems, as well as investigating operational limits to enable the effective development of new TVWS services. Additionally, it consolidated and compared the results of practical tests examining the coexistence between DTV and TVWS signals used as interference and allocated on channels n−1, n+1, n−2, and n+2 conducted in laboratory and field environments, covering different distances.

Laboratory tests involved assessments on the bench and in a controlled environment, analyzing power curves of DTV versus PR_avg_. The results evidenced that the PR varies according to the interfering technology and the spectral positioning of the TVWS signal to the DTV. The lowest PR recorded was −33.24 dB for 5G-RANGE in GFDM on channel n+1, indicating low OOBE and less impact on DTV reception. The highest PR observed was −25.85 dB for 5G NR on channel n+1, demonstrating a greater potential for interference. In addition, it was found that the simultaneous presence of interferers on channels n−1 and n+1 resulted in an average increase of 3 dB in PR, reinforcing that multiple sources of interference can significantly affect DTV reception. Overall, the results demonstrated that the TVWS signal power could be significantly higher than that of DTV without causing perceptual and visible artifacts or errors on decoded video.

Field tests at short distances confirmed these conclusions, highlighting the effective transmission capability of TVWS and that the spectral position of the interference and the receiver model significantly influence the PR. In these tests, it was evident that the performance of the receivers also varied significantly when analyzing the interference on channels n+2 and n−2. STB 1 presented a PR approximately 10 dB higher than the other models, indicating a lower capacity to reject interference. In other words, STBs 2 and 3 demonstrated greater resilience, supporting interfering signals with up to 10 times higher power in n+2 and n−2.

Furthermore, long-distance tests revealed that increasing the TVWS power to up to four times the regulated level (6 dB above the regulatory limit) did not disrupt DTV signals. This result indicates that adjustments in the regulation could allow greater flexibility without compromising digital television reception. The results highlighted that the current regulatory power limitation, set at 1 Wp, compromises the coverage range, particularly in applications that require long distances. Compared to previous tests, which aimed to verify long-range coverage while maintaining support for a throughput of 100 Mbps and in which there were no power restrictions, the system was able to achieve 102 Mbps at 50 km distance with 120.2 Wp, demonstrating that with higher power, it is possible to provide reliable connectivity for large regions. In contrast, the current tests showed that with 1 Wp, the maximum throughput was 96 Mbps at 14.7 km from the base station, while increases to 2 Wp and 4 Wp made it possible to reach 120 Mbps. In the most extreme scenario, at MS_Pedralva (38.34 km from the BS), the system operated at its limit with 1 Wp, reaching a throughput of only 16 Mbps for a 24 MHz BW. However, increasing the power to 8 Wp allowed for a tripling of the throughput.

It is important to highlight that the long-distance tests were conducted considering LOS communication to highlight the impact of transmission power limitation on the maximum coverage of the system. In this scenario, it was possible to understand the effects of power restriction more clearly, eliminating additional variables of NLOS communication. The tests were carried out in a rural environment, as this is the key scenario for 5G-RANGE, and is also the basis of previous tests used as a reference. The analysis of the tests shows that, in open-field scenarios, the technology can reach greater distances with higher powers, while in urban and mountainous areas, propagation could probably be affected by physical and topographical barriers. However, tests in scenarios other than LOS communication were not carried out. Therefore, it is not known for sure what the implications of the regulation would be in NLOS conditions, where natural and artificial obstacles could significantly impact signal propagation, and, therefore, power limitation could be even more detrimental to TVWS services.

The potential for practical application of TVWS in the remote and rural areas of Brazil is significant, especially for expanding internet access in low-density regions where traditional telecommunications infrastructure is limited or non-existent. By offering long-range, low-cost connectivity, TVWS has enormous potential to enable everything from essential services, such as health and education, to advanced applications, such as smart agriculture and public safety, with remote sensing applications for precision agriculture, connectivity for autonomous agricultural machinery, and video surveillance for rural security, among others. These results reinforce that regulatory adjustments, such as a controlled increase in the power limit and optimization of OOBEs, could significantly expand the potential use of the technology. By limiting OOBEs, the regulation would allow the adoption of more efficient modulation techniques in terms of spectral allocation, which could serve as technological differentiators between different standards and equipment manufacturers. Finally, imposing stricter requirements on DTV receivers can ensure greater robustness to interference, enabling more efficient use of the spectrum without compromising the quality of television broadcasting. In any case, it is important to emphasize that, even with current restrictions, TVWS networks can offer high-quality services and robust support for various applications in rural and remote areas.

Future work may explore analyses in urban, mountainous, and densely vegetated environments, where signal propagation can be significantly affected by physical obstacles and multipath reflections, to broaden the understanding of the feasibility of TVWS in different scenarios. Tests in NLOS conditions will allow us to evaluate how power restriction impacts coverage and signal quality in situations where there is no direct line of sight between transmitter and receiver.

This work contributes substantially to the technical and regulatory understanding of TVWS technology in Brazil, providing a solid foundation for the development of public policies and industrial standards that encourage its adoption. With these guidelines, TVWS systems are expected to play a crucial role in the digital transformation of rural and urban areas, promoting digital inclusion, environmental sustainability, and the advancement of smart networks.

## Figures and Tables

**Table 1:** 5G-RANGE main parameters.

	Numerologies					
ID	0	1	2	3	4	5
Sample rate (MHz)	30.72					
Waveform	GFDM (OFDM as a special case)					
Subcarrier spacing (MHz)	1.875	3.75	7.5	15	30	30
Number of subcarriers	16,384	8192	4096	2048	1024	1024
Number of active subcarriers (for BW 23.76 MHz)	12,672	6336	3168	1584	792	792
GFDM subsymbols	4	4	4	4	4	2
CP duration (μs)	141.7	70.8	35.4	17.7	8.9	4.4
CS duration (μs)	25	12.5	6.25	3.13	1.56	0.78
Symbol duration (μs)	2133.3	1066.7	533.3	266.7	133.7	66.7
Symbol duration (μs)	2300	1150	575	287	147.75	71.9
Modulation	QPSK up to 256-QAM	QPSK up to 256-QAM	QPSK up to 256-QAM	QPSK up to 256-QAM	QPSK up to 256-QAM	QPSK up to 256-QAM
CP protection (km)	230	120	60	30	14	7
Mobility supported (km/h)	7	15	30	60	120	240

**Table 2:** Settings for the generation of interfering signals.

Interference Signal	Settings	BW (MHz)	Input Receiver Power (dBm)
5G-RANGE	GFDM and OFDM modulations	6	13
5G NR	Test mode: NR-FR1-TM1.1—ETSI TS 138 141-1 [4]	5	9
4G/LTE	Test mode: E-TM1.1—ETSI TS 136 141 [3]	5	9

**Table 3:** Tests with simultaneous interferences on channels n−1 and n+1.

Interference on Channels *n* + 1 and *n* − 1	PR (dB) System: 4G	PR (dB) System: 5G-RANGE
Non-simultaneous	−29.1	−33.1
Simultaneous 4G (n−1) and 5G-RANGE (n+1)	−26.0	−30.2

**Table 4:** Frequency allocations for short-distance field tests.

**UHF Channel**	26	27	28	29	30
**Frequencies [MHz]**	542 to 548	548 to 554	554 to 560	560 to 566	556 to 572
**TVWS Transmission—5G-RANGE**	-	-	Yes	-	-
**DTV Transmission (One Channel at a Time)**	Yes	Yes	-	Yes	Yes
**Interference Test Channel**	n+2	n+1	-	n−1	n−2
***P*_RX_ DTV**	∼−77 dBm				

**Table 5:** Test results with interference n−1 and n+1 at distances of 15 m and 50 m.

DTV	Set-Top Box	1	2	3	1	2	3
Channel	Link Length [m]	15			50		
	*P*_TX_ TVWS (CH28) [Wp]	0.030	0.030	0.045	0.030	0.030	0.040
27	*P*_RX_ DTV (CH27) [dBm]	−77.1	−77.0	−76.9	−77.0	−77.0	−77.0
	*P*_RX_ TVWS (CH28) [dBm]	−43.2	−43.1	−41.4	−44.3	−44.3	−43.3
Interf.	PR_n+1_ [dB]	−33.9	−33.9	−35.5	−32.7	−32.7	−33.7
n+1	ACPR_UPPER_ [dB]	33.9	33.9	35.5	32.7	32.7	33.7
	ACPRlower [dB]	−15.9	−16.1	−16.1	−16.1	−16.1	−16.1
	*P*_TX_ TVWS (CH28) [Wp]	0.030	0.030	0.040	0.030	0.045	0.054
29	*P*_RX_ DTV (CH29) [dBm]	−76.8	−76.8	−76.7	−76.8	−76.7	−76.7
	*P*_RX_ TVWS (CH28) [dBm]	−42.6	−42.6	−41.8	−43.7	−42.2	−41.7
Interf.	PR_n−1_ [dB]	−34.2	−34.2	−34.9	−33.1	−34.5	−35.0
n−1	ACPR_UPPER_ [dB]	−16.2	−16.2	−16.3	−16.3	−16.3	−16.4
	ACPRlower [dB]	34.2	34.2	34.9	33.1	34.5	35.0

**Table 6:** Test results with interference n−2 and n+2 at distances of 15 m and 50 m.

DTV	Set-Top Box	1	2	3	1	2	3
Channel	Link Length [m]	15			50		
	P_TX_ TVWS (CH28) [Wp]	0.040	3.55	3.55	0.79	7.94	3.98
26	*P*_RX_ DTV (CH26) [dBm]	−76.8	−76.7	−76.7	−76.8	−76.8	−76.8
	*P*_RX_ TVWS (CH28) [dBm]	−31.6	−22.1	−22.1	−29.9	−19.8	−22.7
Interf.	PR_n+2_ [dB]	−45.2	−54.6	−54.6	−46.9	−57.0	−54.1
n+2	ACPR_UPPER_ [dB]	−6.0	1.0	1.0	−6.5	8.3	1.6
	ACPRlower [dB]	−16.1	−16.1	−16.1	−16.2	−15.7	−16.0
	*P*_TX_ TVWS (CH28) [Wp]	0.32	3.55	3.55	0.40	7.94	3.98
30	*P*_RX_ DTV (CH30) [dBm]	−77.2	−77.1	−77.1	−77.1	−77.6	−77.0
	*P*_RX_ TVWS (CH28) [dBm]	−32.2	−22.2	−22.2	−31.9	−20.3	−22.6
Interf.	PR_n−2_ [dB]	−45.0	−54.9	−54.9	−45.2	−56.4	−54.4
n−2	ACPR_UPPER_ [dB]	−15.8	−15.5	−15.5	−16.0	−14.2	−15.9
	ACPRlower [dB]	−6.6	−0.2	−0.2	−6.6	8.2	0.1

**Table 7:** Predicted powers for the TVWS BS.

Total *P*_peak_ [Wp]	Total *P*_peak_ [dBm]	PAPR [dB]	Total *P*_avg_ [dBm]	*P*_avg_ to Each Antenna (MIMO 2 × 2) [dBm]
1	30	10	20	17
2	33	10	23	20
4	36	10	26	23

**Table 8:** Coexistence tests results in MS_Loteamento with LNA turned on and off.

	*P*_TX_ TVWS (CH28) [Wp]	1	2	4	1	2	4
DTV	Filter_RX_ (CH28)	Yes			Yes		
Channel	LNA_RX_	Off			On		
	*P*_RX_ DTV (CH27) [dBm]	−77.5	−77.5	−77.4	−77.5	−77.4	−77.5
	*P*_RX_ TVWS (CH28) [dBm]	−80.2	−76.5	−73.7	−57.1	−53.2	−50.5
27	PR_(n+1)_ [dB]	2.7	−1.0	−3.7	−20.4	−24.2	−27.0
	ACPR_UPPER_ [dB]	−2.7	1.0	3.7	20.4	24.2	27.0
Interf.	ACPR_LOWER_ [dB]	−16.0	−16.0	−16.0	−16.0	−15.9	−15.9
n+1	*P*_RX_ INT. (CH30) [dBm]	-	-	-	−84.1	−84.1	−84.1
	DTV STB Status 1	OK	OK	OK	OK	OK	OK
	DTV STB Status 2	OK	OK	OK	OK	OK	OK
	DTV STB Status 3	OK	OK	OK	OK	OK	OK
	*P*_RX_ DTV (CH29) [dBm]	−77.1	−77.0	−77.0	−77.0	−77.0	−77.0
	*P*_RX_ TVWS (CH28) [dBm]	−78.9	−75.1	−72.3	−55.2	−51.5	−48.7
29	PR_(n−1)_ [dB]	1.8	−1.9	−4.7	−21.8	−25.5	−28.3
	ACPR_UPPER_ [dB]	−16.4	−16.2	−16.2	−8.9	−8.9	−8.9
Interf.	ACPR_LOWER_ [dB]	−1.8	1.9	4.7	21.8	25.5	28.3
n−1	*P*_RX_ INT. (CH30) [dBm]	-	-	-	−85.9	−85.9	−85.9
	DTV STB Status 1	OK	OK	OK	OK	OK	OK
	DTV STB Status 2	OK	OK	OK	OK	OK	OK
	DTV STB Status 3	OK	OK	OK	OK	OK	OK

**Table 9:** Additional test results with n−1 interference at the MS_Loteamento location.

	*P*_TX_ TVWS (CH28) [Wp]	6.3	8	10
DTV	Filter_RX_ (CH28)	Yes		
Channel	LNA_RX_	On		
	*P*_RX_ DTV (CH29) [dBm]	−76.9	−76.9	−77.0
	*P*_RX_ TVWS (CH28) [dBm]	−46.7	−45.8	−45.1
29	PR_(n−1)_ [dB]	−30.2	−31.1	−31.9
	ACPR_UPPER_ [dB]	−7.6	−8.9	−7.6
Interf.	ACPR_LOWER_ [dB]	30.2	31.1	31.9
n−1	*P*_RX_ INT. (CH30) [dBm]	−84.5	−85.8	−84.6
	DTV STB Status 1	OK	Interm.	NOK
	DTV STB Status 2	OK	OK	Interm.
	DTV STB Status 3	OK	OK	OK

**Table 10:** Additional test results with n+1 and n−1 interference at the MS_Loteamento location.

	*P*_TX_ TVWS (CH28) [Wp]	4		
DTV	Filter_RX_ (CH28)	No		
Channel	Att. after LNA_RX_ (dB)	4	3	2
	LNA_RX_	On		
	*P*_RX_ DTV (CH29) [dBm]	−76.9	−76.8	−76.8
29	*P*_RX_ TVWS (CH28) [dBm]	−51.2	−50.0	−49.0
	PR_(n+1)_ [dB]	−30.1	−31.1	−31.8
	PR_(n−1)_ [dB]	−25.7	−26.8	−27.8
Interf.	ACPR_UPPER_ [dB]	30.1	31.1	31.8
n+1	ACPR_LOWER_ [dB]	25.7	26.8	27.8
e	*P*_RX_ INT. (CH30) [dBm]	−46.8	−45.7	−45.0
n−1	DTV STB Status 1	OK	NOK	NOK
	DTV STB Status 2	OK	OK	NOK
	DTV STB Status 3	OK	OK	OK

**Table 11:** Results of reception tests for the 5G-RANGE (TVWS) system at the locations MS_Loteamento and MS_Delta Black.

Local MS	*P*_RX Ant._ [dBm]	SNR [dB]	MCS	Bitrate [Mbps]	Bitrate_4 Ch_ [Mbps]
Loteamento	−64.7	34.1	256-QAM—Polar 5/6	30	120
DeltaBlack	−68.6	32.8	256-QAM—Polar 5/6	30	120

**Table 12:** Results of reception tests for the 5G-RANGE (TVWS) system at the location MS_CafezalManoela.

*P*_TX_ [Wp]	*P*_RX Ant._ [dBm]	SNR [dB]	MCS	Bitrate [Mbps]	Bitrate_4 Ch_ [Mbps]
1	−76.6	25.6	256-QAM—Polar 2/3	24	96
2	−73.1	28.84	256-QAM—Polar 5/6	30	120
4	−70.3	31.15	256-QAM—Polar 5/6	30	120

**Table 13:** Results of reception tests for the 5G-RANGE (TVWS) system at the location MS_Pedralva.

*P*_TX_ [Wp]	*P*_RX Ant._ [dBm]	SNR [dB]	MCS	Bitrate [Mbps]	Bitrate_4 Ch_ [Mbps]
1	−83.9	7.69	QPSK—Polar 2/3	4	16
2	−81.3	11.47	QPSK—Polar 3/4	7	28
4	−79.2	14.31	16-QAM—Polar 2/3	12	48
8	−75.8	17.19	16-QAM—Polar 3/4	13	52

## Data Availability

Data available on reasonable request.

## Abbreviations

The following abbreviations are used in this manuscript:

4G	Fourth generation
5G	Fifth generation
5G NR	5G New Radio
5G-RANGE	Remote Area Access Network for the Fifth Generation
ABNT	Associação Brasileira de Normas Técnicas
ACLR	Adjacent Channel Leakage–Power Ratio
ACPEs	Adjacent Channel Power Emissions
ACPR	Adjacent Channel Power Ratio
Anatel	Agência Nacional de Telecomunicações
ANE	Agencia Nacional del Espectro
BER	Bit Error Rate
BS	Base Station
BTS	Broadcast Transport Stream
BW	Bandwidth
DPD	Digital Pre-Distortion
DTV	Digital Television
DVRG	Digital Video Recorder Generator
EIRP	Effective Isotropic Radiated Power
ETSI	European Telecommunications Standards Institute
FCC	Federal Communications Commission
FEC	Forward Error Correction
F-OFDM	Filtered Orthogonal Frequency Division Multiplexing
GFDM	Generalized Frequency Division Multiplexing
HAAT	Height Above Average Terrain
ICASA	Independent Communications Authority of South Africa
IEEE	Institute of Electrical and Electronics Engineers
Inatel	Instituto Nacional de Telecomunicações
ISDB-T	Integrated Services Digital Broadcasting–Terrestrial
ITU	International Telecommunication Union
LNA	Low-Noise Amplifier
LTE	Long-Term Evolution
LTE-A	LTE-Advanced
MCS	Modulation and Coding Scheme
MIMO	Multiple-Input and Multiple-Output
MS	Mobile Station
NBR	Norma Brasileira
Ofcom	Office of Communications
OFDM	Orthogonal Frequency Division Multiplexing
OOBE	Out-of-Band Emission
PAPR	Peak-to-Average Power Ratio
PR	Protection Ratio
QAM	Quadrature Amplitude Modulation
QEF	Quasi Error Free
QPSK	Quadrature Phase Shift Keying
RF	Radiofrequency
RMS	Root Mean Square
SEFICE	Serviço Especial para Fins Científicos e Experimentais
SNR	Signal-to-Noise Ratio
STB	Set-Top Box
TOV	Threshold of Visibility
TVWS	TV White Space
UFC	Universidade Federal do Ceará
UK	United Kingdom
USA	United States of America
UHF	Ultra High Frequency
VHF	Very High Frequency
VPN	Virtual Private Network
WSD	White Space Device
WSDB	White Space Database
